# CXCR5^+^TIM-3^-^PD-1^+^ stem-like cytotoxic CD8^+^ T cells: elevated in chronic rhinosinusitis and associated with disease severity

**DOI:** 10.3389/fimmu.2024.1295309

**Published:** 2024-02-15

**Authors:** Zhichen Liu, Zixuan Zhao, Huanxia Xie, Ning Lu, Jisheng Liu, Qingqing Jiao

**Affiliations:** ^1^ Department of Ear, Nose, and Throat, The First Affiliated Hospital of Soochow University, Suzhou, China; ^2^ The First Clinical Medicine School, Suzhou Medical College, Suzhou University, Suzhou, China; ^3^ Department of Dermatology, The First Affiliated Hospital of Soochow University, Suzhou, China

**Keywords:** chronic rhinosinusitis, PD-1^+^, CXCR5^+^, TIM-3^-^, stem-like CD8^+^ T cells

## Abstract

**Background:**

Chronic rhinosinusitis (CRS) is a chronic inflammatory disease with an autoimmune background. Altered expression levels of T cell immunoglobulin and mucin-domain containing-3 (TIM-3), C-X-C chemokine receptor type 5 (CXCR5), and programmed cell death protein 1 (PD-1) are implicated in the progression of inflammatory and autoimmune diseases. Moreover, CXCR5^+^TIM-3^-^PD-1^+^ stem-like cytotoxic T cells function as memory stem cells during chronic disease processes and retain cytotoxicity-related gene networks.

**Objectives:**

To explore the expressions of CXCR5, TIM-3, and PD-1 on T cells and their correlation with clinical parameters in CRS.

**Methods:**

Flow cytometry was used to assess the expressions and co-expressions of CXCR5, TIM-3, and PD-1 on T cells in the tissues of the paranasal sinus and peripheral blood of patients with CRS as well as healthy controls. Immunofluorescence was used to assess the co-localization of TIM-3, CXCR5, and PD-1 with T cells. The disease severity of our patients with CRS was evaluated using the Lund-Mackay score. A complete blood count was also performed for the patients with CRS.

**Results:**

Expression levels of CXCR5 and PD-1 on T cells were significantly increased in the nasal tissues of patients with CRS. Compared with those in healthy controls, patients with CRS had high percentages of CXCR5^+^TIM-3^-^PD-1^+^ CD8^+^ and CD4^+^ T cells in nasal tissues, while no significant difference was observed in peripheral blood levels. Patients with CRS had a higher density of nasal CXCR5+TIM-3-PD-1+ T cells than that in healthy controls. CXCR5^+^TIM-3^-^PD-1^+^ CD8^+^ T cell levels in the nasal polyps of patients with CRS were negatively correlated with the patients’ Lund-Mackay scores. The levels of CXCR5^+^TIM-3^-^PD-1^+^ T cells in nasal tissues were also negatively associated with disease duration and positively associated with the chronic inflammatory state of CRS.

**Conclusions:**

The level of CXCR5^+^TIM-3^-^PD-1^+^ stem cell-like T cells, especially CXCR5+TIM-3-PD-1+ CD8+ T cells, is increased in CRS. Therefore, inducing CXCR5^+^TIM-3^-^PD-1^+^ T cell exhaustion may be an effective immunotherapy for CRS.

## Introduction

Chronic rhinosinusitis (CRS) is a multifactorial, chronic inflammatory disease of the upper respiratory tract. The disease is characterized by nasal blockage, obstruction, congestion, or nasal discharge, and currently affects 5–12% of the world’s population (1). Patients with nasal polyps tend to have a high disease burden and high recurrence rates (1). The pathogenesis of CRS involves multiple factors, including infection and immunity, and the process combines the characteristics of both chronic inflammatory and autoimmune diseases (1–3).

As is known, the inflammatory response is present in a wide range of chronic diseases. Simultaneously, autoimmunity drives the onset and progression of the inflammatory response (4, 5). Inflammatory responses are mediated by a variety of cells and cytokines that exert destructive effects on tissues (1, 2). The immune dysregulation underlying CRS is closely associated with T-cell dysfunction; thus, discovering a T-cell subset that plays a central role in this immune dysregulation is critical to identifying potential therapeutic targets in patients with CRS (1, 6).

In recent years, a highly functional subpopulation of stem-like T cells has been identified in tumors and viral infections, retaining both stem cell-like self-renewal and multipotency as well as a gene network for cytotoxicity (7–10). These cells have been characterized as CXCR5^+^TIM-3^-^PD-1^+^CD8^+^ T cells and demonstrate a proliferative burst upon blockade of the programmed cell death protein1 (PD-1) receptor, which has been significant in the development of PD-1-targeted therapy for chronic viral infections and tumors (7, 11, 12). This CD8^+^ T cell subset exhibits self-renewal and differentiation in chronic viral infections and tumors (7, 11). This generates terminally exhausted CD8^+^ T cells in lymphoid and non-lymphoid tissues, including CXCR5^+/-^ and TIM3^+/-^ phenotypes (7, 11). Among them, CXCR5^-^CD8^+^ T cells express high levels of effector molecules (11). Compared with CXCR5^-^TIM-3^+/-^ cells, CXCR5^+^TIM-3^-^ cells evince synergistic production of multiple effector molecules (7). Additionally, CXCR5+TIM-3- cells can maintain rapid effector function and multifunctionality *in vitro (* 7). The remarkable pluripotency and cytotoxicity of these stem-like T cells have attracted the attention of several researchers. However, no studies have demonstrated changes in this cell subpopulation in non-viral chronic inflammatory and autoimmune diseases.

In previous studies, C-X-C chemokine receptor type 5 (CXCR5) has been demonstrated to play a vital role in the recruitment and activation of immune cells and the regulation of adaptive immune responses. CXCL13/CXCR5 signaling regulates cancer and immune cells and is involved in inflammation, autoimmunity, and tumor development (13, 14). In addition, T cell immunoglobulin and mucin-domain containing-3 (TIM-3) and PD-1 checkpoint molecular receptors may be involved in the mechanism of immune dysregulation of T cells underlying autoimmune diseases (15–18). PD-1 exhibits an anti-inflammatory role and counteracts T cell-mediated inflammatory responses in various inflammatory diseases. Hence, the knockdown or blockade of PD-1 induces or exacerbates inflammatory responses (19–21). In the lung inflammation and fibrosis model, anti-TIM-3-treated mice displayed more severe inflammation and peribronchial fibrosis than that exhibited by control mice (22). In conclusion, CXCR5, TIM-3, and PD-1 receptors play vital roles in immune-related diseases, but no study has yet elucidated whether CXCR5^+^TIM-3^-^PD-1^+^ T cells are involved in the development of CRS.

In light of the fundamental role of T cell dysfunction in CRS, the importance of CXCR5, TIM-3, and PD-1 molecules in inflammatory and autoimmune diseases, and the unique function of stem-like cytotoxic CD8+ T cells, the present study investigated the expression of CXCR5^+^TIM-3^-^PD-1^+^ T cells in patients with CRS for the first time. In this study, we investigated the expression of CXCR5, TIM-3, and PD-1 molecules and their co-expression levels on T cells in patients with CRS to explore the involvement of CXCR5^+^TIM-3^-^PD-1^+^ T cells in the disease process of CRS. We also described the clinical significance of CXCR5^+^TIM-3^-^PD-1^+^ T cells in CRS and addressed the potential mechanisms involved in the pathogenesis of CRS.

## Materials and methods

### Study participants

A total of 20 patients with CRS in the Department of Otolaryngology-Head and Neck Surgery of the First Affiliated Hospital of Soochow University were enrolled according to the guidelines of the European Position Paper on Rhinosinusitis and Nasal Polyps 2020 (1) (**Table 1**). Patients with other infectious, inflammatory, and immunologic diseases were excluded. The control group consisted of nine healthy individuals with no immune-related or chronic diseases (**Table 1**). This study was approved by the Ethics Committee of the First Affiliated Hospital of Soochow University (No. (2023) 056). All participants provided written informed consent following the Helsinki Declaration of the World Medical Association.

**Table 1:** Demographic and clinical profile of the subjects involving in the study.

Sample (n, male%)	CRS (n=20)			HC (n=9)	
	Mucosa (12, 75%)	NP (14, 79%)	PB (20, 60%)	Mucosa (4, 75%)	PB (9, 66%)
**Age (years, mean±SD)**	44.17±11.16	44±11.16	46.82±12.29	34.75±10.72	39.2±11.5
**Duration (months, mean±SD)**	20.71±33.54	25.82±39.73	21.75±33.76	/	/
**Lund-Mackay score (mean±SD)**	8.08±2.61	10.54±2.63	8.44±3.35	/	/
**CRSwNP, n (%)**	9 (75%)	14 (100%)	17 (85%)	/	/
**Comorbidity, n (%)**					
**Asthma**	0 (0)	1 (7%)	1 (5%)	/	/
**Aspirin intolerance**	0 (0)	1 (7%)	1 (5%)	/	/

HC, healthy control; NP, nasal polyp; PB, peripheral Blood; SD, standard deviation; CRSwNP, chronic rhinosinusitis with nasal polyp.

### Clinical assessment

For patients with CRS, all individuals were evaluated by a rhinologist, and demographic and clinical data (gender, age, disease duration, symptoms) were obtained. The paranasal sinus Lund-Mackay (LM) score was assessed by computed tomography (CT) scan. The LM score is commonly used to evaluate the severity of CRS (23). High scores represent severe manifestations of inflammation. A complete blood count was performed on peripheral blood samples to obtain additional clinical parameters. Demographic data of healthy controls were also collected. We obtained peripheral blood and paranasal sinus tissue samples from both patients and healthy controls. These paranasal sinus tissues were further divided into two categories: paranasal sinus mucosa and nasal polyps. The paranasal sinus mucosa was obtained from the middle turbinate and uncinate processes of the participants. Moreover, nasal polyps are diseased polypoid tissue in the patient’s paranasal sinuses; healthy donors do not have nasal polyps.

### Preparation of tissue single-cell suspensions

As previously described (24), paranasal sinus tissues were mechanically separated into small pieces by scissors and digested with 0.33 mg/mL DNase (Sigma-Aldrich) and 0.27 mg/mL Liberase TL (Roche) in serum-free Roswell Park Memorial Institute 1640 for 25 minutes at 37°C, and then filtered through a 70 m cell strainer to make a single-cell suspension.

### Flow cytometry analysis

**Figure 1:**
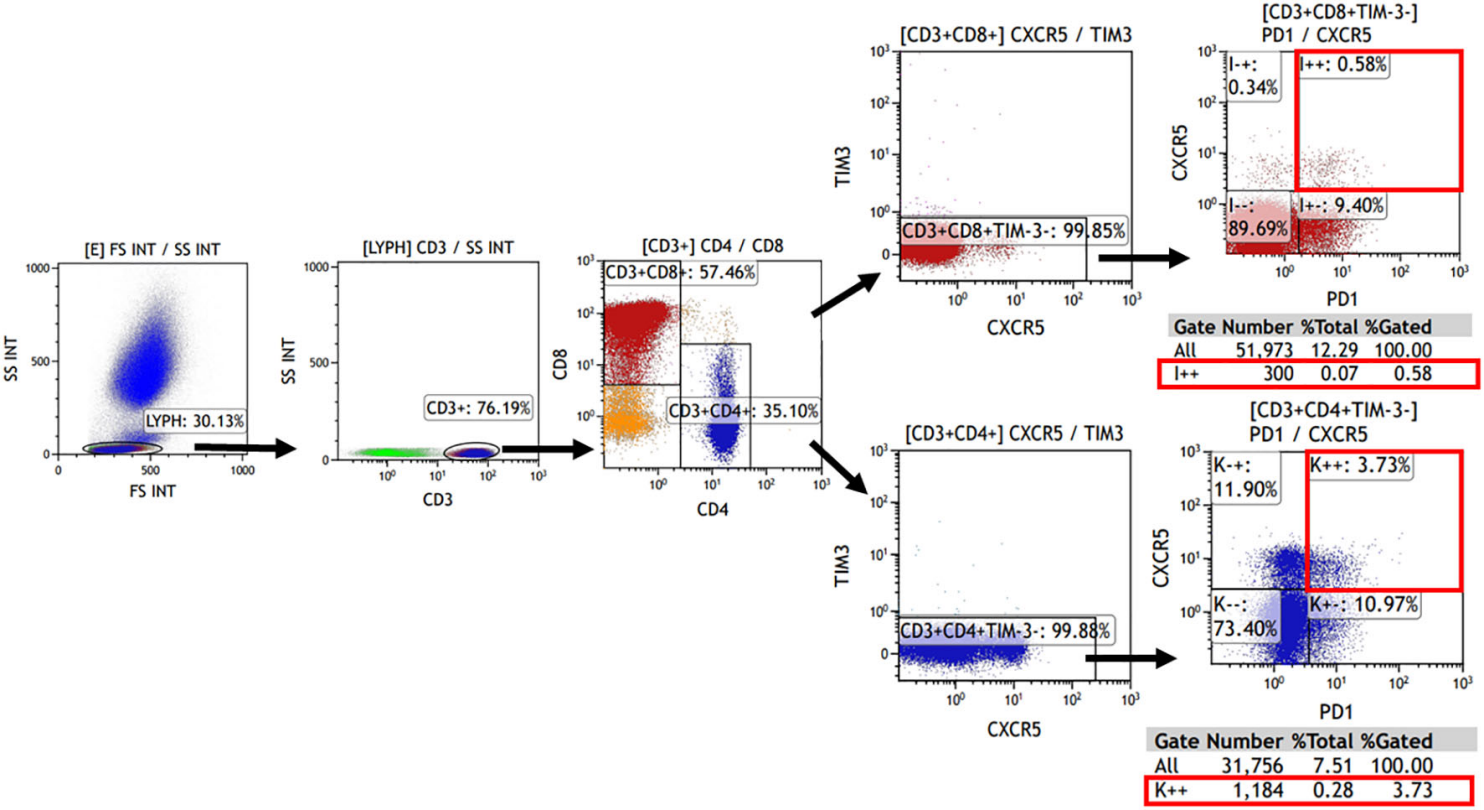
The gating strategy for flow cytometry.

The expression of TIM-3, CXCR5, and PD-1 on CD4^+^ or CD8^+^ cells was evaluated using flow cytometry. Tissue single-cell suspensions, as well as peripheral blood (after lysing erythrocytes), were used for analysis. Cells were briefly re-suspended in Hank’s balanced salt solution with 1% fetal bovine serum. Additionally, 100 μL of the cell suspension was incubated with fluorochrome-conjugated monoclonal antibodies for 20 minutes at 4°C in the dark. All antibodies were obtained from Biolegend (San Diego, CA, USA) with the following details: Brilliant Violet 510™ anti-human CD3 (Cat. No. 317331), PerCP/Cyanine5.5 anti-human CD4 (Cat. No. 317427), FITC anti-human CD8 (Cat. No. 344703), Brilliant Violet 421™ anti-human CD366 (Tim-3) (Cat. No. 345007), PE/Cy7 anti-human CD185 (CXCR5) (Cat. No. 356923), APC anti-human CD279 (PD-1) (Cat. No. 329907). Samples were then analyzed using Kaluza Analysis Software (Beckman, United States). The analysis involved initially gating CD3+ cells in the lymphocyte population, followed by the detection of CD4+ and CD8+ cells within the CD3+ lymphocytes. Subsequently, the TIM-3- and CXCR5+PD-1+ subpopulations were detected separately. The corresponding gating strategies are displayed in **Figure 1**. Single color compensation controls were performed to determine the amount of spillover of each fluorochrome to the other channels for compensation analysis. The results confirmed the percentage and the number of CD4^+^ or CD8^+^ CXCR5^+^TIM-3^-^PD-1^+^ T cells in the sample.

### Immunofluorescence staining

Paranasal sinus tissues of the participants were embedded in paraffin and prepared into 4 μm thick sections. After paraffin removal, the deparaffinized sections were heat-treated in citrate repair buffer (pH 6.0), followed by the addition of drops of 3% hydrogen peroxide to remove endogenous peroxidase. The sections were treated with goat serum blocking solution for 20 minutes and then reacted with the following primary antibodies for 1 hour for immunofluorescence staining: CD3 (1:1500, ab16669, Abcam), CXCR5 (1:5000, ab254415, Abcam), TIM-3 (1:1000, ab241332, Abcam), PD-1 (1:500, ab237728, Abcam). After washing, the sections were incubated with a secondary antibody (PV-6001, ZSGB-BIO) for 20 minutes. Subsequently, a rapid reaction buffer (1:100, FFBN45, DMK) was added and the sections were incubated at room temperature for 5 minutes for color development. The antibody complexes were removed by microwave treatment and the markers were counterstained. The sections were counterstained with 4′,6-diamidino-2-phenylindole at room temperature for 5 minutes. The sections were finally closed with the antifade mounting medium. After completion of staining, the sections were imaged using the TissueFAXS whole-slide scanning system (TissueGnostics, USA) and analyzed using the TissueQuest image analysis software (TissueGnostics, USA) to obtain accurate cell counts and densities.

### Single-cell sequencing analysis

In this manuscript, we analyzed the raw data from the single-cell database. The accession number of the raw sequencing data of human samples is Genome Sequence Archive HRA000772.

**Figure 2:**
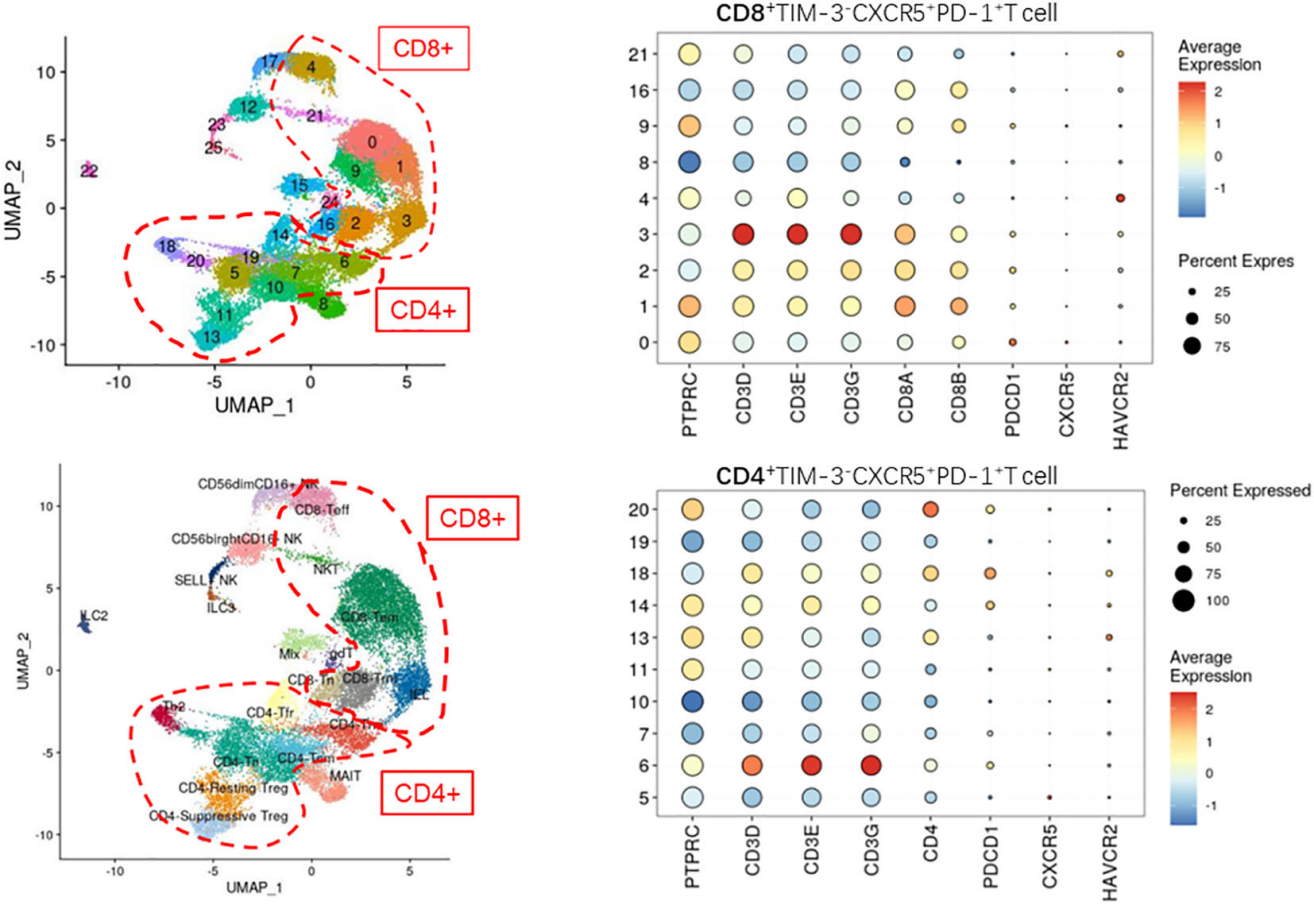
Defining the CD4+/CD8+CXCR5+TIM-3-PD-1+ T cell subpopulation by single-cell sequencing (not found).

Cell Ranger (version 5.0) was applied to filter low-quality reads, align reads to the human reference genome (GRCh38), assign cell barcodes, and generate the unique molecular identifier (UMI) matrices. R software (version 3.6.1) was used to analyze the output gene expression matrices with the Seurat package (version 3.2.0). All samples were merged into one Seurat object using the merge function. We subsequently filtered out cells with less than 200 genes detected, cells with less than 500 UMI counts detected, and cells with more than 10% mitochondrial UMI counts. Dimension reduction and unsupervised clustering were performed according to the standard workflow in Seurat. SCTransform function was applied to normalize and find highly variable genes (hvgs) within the single-cell gene expression data. Mitochondrial, dissociation-induced, and Human leukocyte antigens genes were removed from hvgs for downstream analysis. Then, the effect of the percentage of mitochondrial gene count was regressed using the SCTransform function with parameter “vars.to.regress=‘percent.mt’”. A principal component analysis (PCA) matrix was calculated using RunPCA with default parameters to reduce noise. Harmony (version 1.0) was applied immediately after PCA to remove batch effects from different samples. Next, Uniform Manifold Approximation Projection and clustering were performed on the “harmony space” to identify clusters. The main immune cell types were annotated based on the expression patterns of differentially expressed genes and well-known cellular markers from the literature. In the first round of unsupervised clustering of all cells, we identified that PTPRC and HBB were co-expressed in some clusters, hence we removed these clusters from the dataset for downstream analysis. Finally, we clustered this subpopulation in CD4^+^ and CD8^+^ T cells with the corresponding genes for CXCR5^+^TIM-3^-^PD-1^+^ cells (**Figure 2** displays the detailed genes).

### Statistical analysis

Statistical analysis was performed using GraphPad Prism 7 software (GraphPad, San Diego, CA). The Shapiro–Wilk normality test was used to determine variable distributions. Student’s t-test and Mann–Whitney U-test were used to compare groups for variables that did or did not fit the normal distribution, respectively. In addition, Spearman’s correlation test was used to determine the linear correlation coefficient. Outliers were excluded from the analysis. P-values less than 0.05 were considered significant.

## Results

### High expression of CXCR5 and PD-1 molecules on T cells in paranasal sinus mucosa of patients with CRS

**Figure 3:**
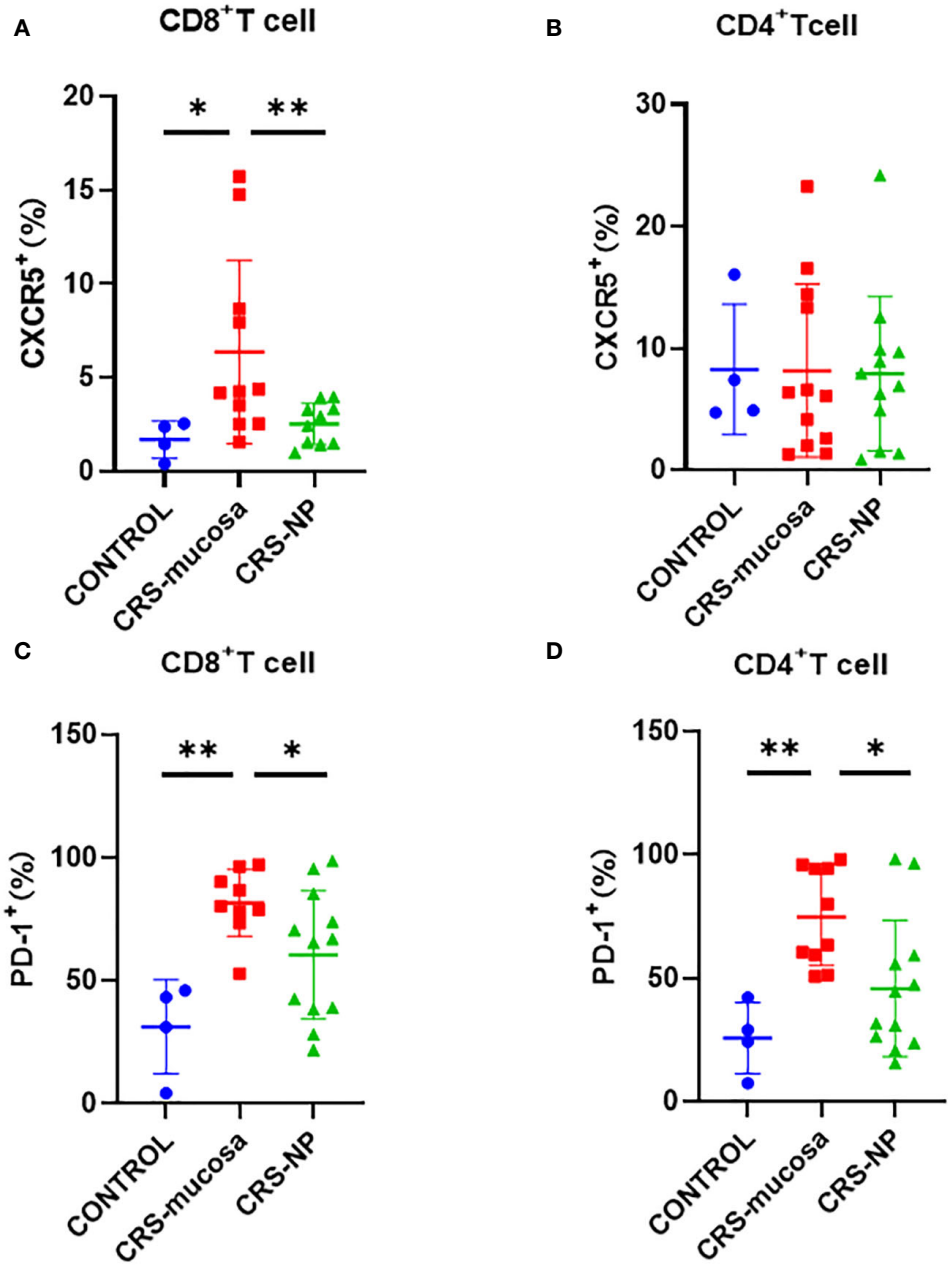
Percentage of CXCR5 and PD-1 molecules among CD4+/CD8+ T cells in different tissues. Percentage of CXCR5+ cells among **(A)** CD8+ and **(B)** CD4+ cells. Percentage of PD-1+ cells in **(C)** CD8+ and **(D)** CD4+ cells. *: p<0.05; **: p<0.01.

The percentage of CXCR5^+^ cells among CD8^+^ T cells was significantly increased in the paranasal sinus mucosa (p=0.018) and nasal polyps (p=0.0079) of patients with CRS than that in healthy controls (**Figure 3A**). However, no significant difference was observed in the proportion of CXCR5^+^ cells among CD4^+^ T cells in the nasal tissues of patients and controls (**Figure 3B**).

Similarly, the percentage of PD-1^+^ cells among CD8^+^ and CD4^+^ T cells was significantly higher in the paranasal sinus mucosa (CD8^+^: p=0.003, **Figure 3C**; CD4^+^: p=0.002, **Figure 3D**) of patients with CRS compared to the percentage in healthy controls. A tendency was also observed for the percentage of PD-1^+^ cells among CD4^+^ and CD8^+^ T cells to be higher in the nasal polyps of patients with CRS patients than in the healthy controls. However, the p-value was not significant (CD8^+^: p=0.17; CD4^+^: p=0.21) (**Figures 3C, D**). Additionally, the percentage of PD-1^+^ cells among both CD8^+^ and CD4^+^ T cells in the paranasal sinus mucosa was significantly higher than that in nasal polyps (**Figures 3C, D**).

### Elevated levels of CXCR5^+^TIM-3^-^PD-1^+^ T cells in the paranasal sinus tissue but not in the peripheral blood of patients with CRS

**Figure 4:**
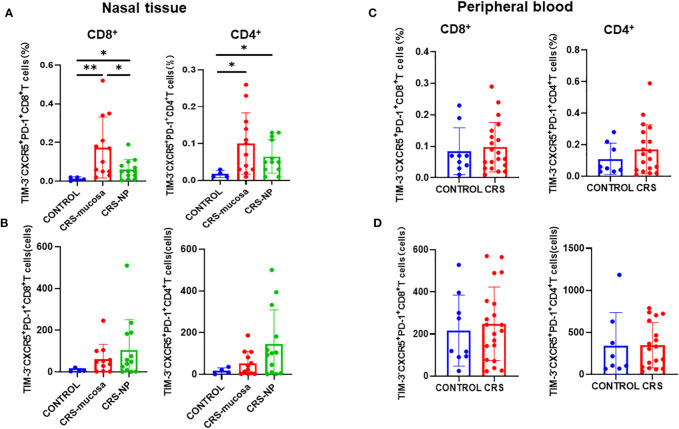
Number of CD4^+^/CD8^+^CXCR5^+^TIM-3^-^PD-1^+^ T cells in tissues and peripheral blood and their percentage of total cells in the sample. **(A)** Percentage of CD4^+^/CD8^+^CXCR5^+^TIM-3^-^PD-1^+^ T cells in paranasal sinus tissue out of the total cells in the sample. **(B)** Number of CD4^+^/CD8^+^CXCR5^+^ TIM-3^-^PD-1^+^ T cells in paranasal sinus tissues. **(C)** Percentage of CD4^+^/CD8^+^ CXCR5^+^TIM-3^-^PD-1^+^ T cells in peripheral blood out of the total cells in the sample. **(D)** Number of CD4^+^/CD8^+^CXCR5^+^TIM-3^-^PD-1^+^ T cells in peripheral blood. CONTROL: healthy control subjects; CRS-mucosa: Paranasal sinus mucosa in chronic rhinosinusitis patients; CRS-NP: Nasal polyps in chronic rhinosinusitis patients; CRS: chronic rhinosinusitis patients. *: p<0.05; **: p<0.01.

The proportion of CXCR5^+^TIM-3^-^PD-1^+^ cells among both CD8^+^ and CD4^+^ T cells was significantly elevated in the paranasal sinus mucosa (CD8^+^: p=0.0027; CD4^+^: p=0.024); and in the nasal polyps (CD8^+^: p=0.042; CD4^+^: p=0.039); of patients with CRS than the proportion in healthy controls (**Figure 4A**). Between the two types of nasal tissues in our patients with CRS, CXCR5^+^TIM-3^-^PD-1^+^ cell levels were significantly higher in the paranasal sinus mucosa than in nasal polyps, especially among the CD8^+^ subpopulation (p=0.046) (**Figure 4A**).

As displayed in **Figure 4B**, in terms of cell numbers, CXCR5^+^TIM-3^-^PD-1^+^ CD8^+^ and CD4^+^ T cells were more abundant in the paranasal sinus tissues of patients with CRS than in those of healthy controls. However, when comparing the two types of tissues, no significant difference was identified in cell count between the nasal polyps and mucosa, although a trend toward high numbers in the nasal polyps could be observed.

**Figure 5:**
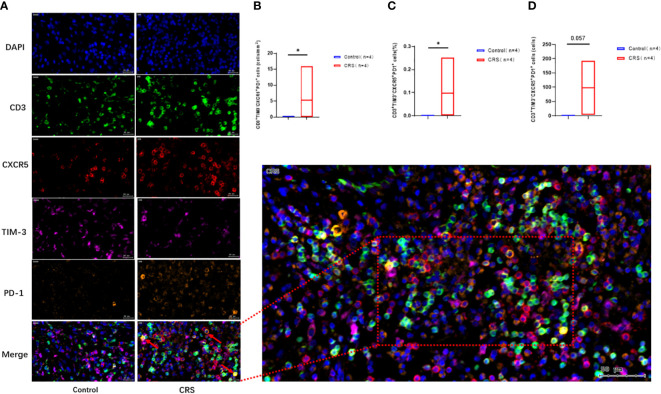
Immunofluorescence analysis of CRS patients and healthy controls. **(A)** Representative immunofluorescence analysis showing co-localization of CD3 (green), CXCR5 (red), TIM-3 (purple), and PD-1 (orange) in the paranasal sinus mucosa of the subjects. Scale bar, 20µm. The arrow labeled CD3^+^CXCR5^+^TIM-3-PD-1^+^ cells. **(B–D)** Immunofluorescence showing the expression of CD3+CXCR5+TIM-3-PD-1+ cells. Box plots show mean and range. *: p<0.05.

Immunofluorescence staining (**Figure 5A**) revealed that CXCR5^+^TIM-3^-^PD-1^+^ T cell density was significantly higher in the paranasal sinus mucosa of patients with CRS than in that of healthy controls (p=0.029, **Figure 5B**). Similar to the flow cytometry results, the proportion of this cell type in immunofluorescence staining was also significantly higher in patients with CRS patients compared to that in healthy controls (p=0.029, **Figure 5C**). The difference in the cell count was not significant (p=0.057, **Figure 5D**), but a trend toward high numbers in patients with CRS could be observed. However, no significant differences were present between patients with CRS and healthy controls when comparing cell counts and proportions in peripheral blood (**Figures 4C, D**).

**Figure 6:**
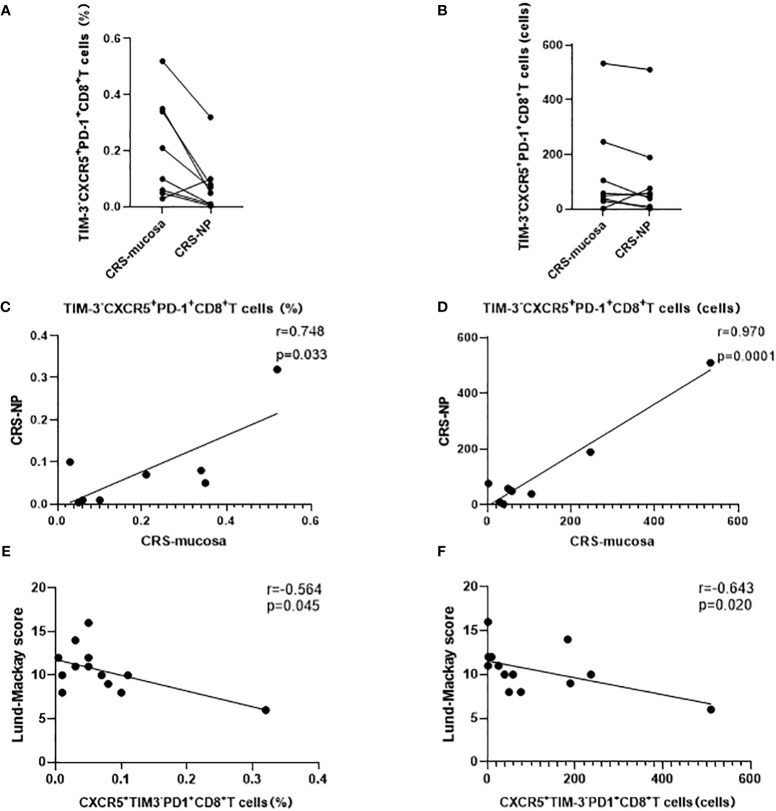
Correlation analysis of CD8^+^ CXCR5^+^TIM-3-PD-1^+^ cells. **(A–D)** Expression and correlation of CD8^+^ CXCR5^+^TIM-3-PD-1^+^ cells in different tissues of the same patient. **(E, F)** Correlation of CD8^+^ CXCR5^+^TIM-3-PD-1^+^ cells with Lund-Mackay score.

In addition, a significant positive correlation was noted between CXCR5^+^TIM-3^-^PD-1^+^ CD8^+^ T cell counts and proportions in paranasal sinus mucosa and nasal polyps from the same patient (percentage: r=0.748, p=0.033; number: r=0.970, p=0.0001; **Figures 6A–D**). This trend was not detected in CXCR5^+^TIM-3^-^PD-1^+^ CD4^+^ T cells (data not displayed).

### CXCR5^+^TIM-3^-^PD-1^+^ CD8^+^ T cell levels negatively correlated with CRS disease severity

Our correlation analysis results demonstrated that the percentage (r=-0.564, p=0.045) and number (r=-0.643, p=0.020) of CXCR5^+^TIM-3^-^PD-1^+^ CD8^+^ T cells in the nasal polyps of patients with CRS were negatively correlated with the LM score, which indicates the severity of disease (**Figures 6E, F**). In contrast, neither the mucosa nor the peripheral blood of patients with CRS patients demonstrated any correlation between CXCR5^+^TIM-3^-^PD-1^+^ T cells and the LM score (**Table 2**).

### CXCR5^+^TIM-3^-^PD-1^+^ T cells associated with disease duration and chronic inflammatory state

As displayed in **Table 2**, the number of CXCR5^+^TIM-3^-^PD-1^+^ T cells in paranasal sinus tissues, including mucosa and nasal polyps, was negatively correlated with the duration of CRS, i.e., in paranasal sinus mucosa, CD4^+^: r=-0.558, p=0.063; CD8^+^: r=-0.599, p=0.043; in nasal polyps, CD4^+^: r=-0.648, p= 0.035; CD8^+^: r=-0.411, p=0.21, respectively.

Leukocyte, neutrophil, lymphocyte, and monocyte levels are suggestive of a chronic inflammatory and infectious state. Accordingly, positive correlations were observed between the levels of CXCR5^+^TIM-3^-^PD-1^+^ T cells in nasal tissues and the levels of leukocytes, neutrophils, lymphocytes, and monocytes (**Table 2**).

**Table 2:** Correlation analysis of CD4^+^/CD8^+^CXCR5^+^TIM-3^-^PD-1^+^ T cells.

	Sinus Mucosa				Nasal Polyps				Peripheral Blood			
	CD4^+^		CD8^+^		CD4^+^		CD8^+^		CD4^+^		CD8^+^	
	Count	PCT(%)	Count	PCT(%)	Count	PCT(%)	Count	PCT(%)	Count	PCT(%)	Count	PCT(%)
**Duration (year)**	r=-0.5583 p=0.0627	r=-0.07381 p=0.8184	**r=-0.5993** **p=0.0426**	r=-0.03163 p=0.9229	**r=-0.6484** **p=0.0352**	r=-0.05287 p=0.8771	r=-0.4110 p=0.2090	r=-0.04157 p=0.9037	r=-0.1425 p=0.5828	r=-0.2667 p=0.2977	r=0.001229 p=0.9981	r=-0.1552 p=0.5485
**Lund-Mackay**	r=0.01645 p=0.9595	r=0.3905 p=0.2351	r=-0.1495 p=0.6609	r=0.1309 p=0.7012	r=-0.02886 p=0.9412	r=-0.009471 p=0.9807	**r=-0.7317** **p=0.0250**	**r=-0.7171** **p=0.0297**	r=0.09433 p=0.7381	r=-0.07952 p=0.7782	r=0.01620 p=0.9562	r=-0.2186 p=0.4337
**WBC#**	r=0.2884 p=0.4196	r=0.4863 p=0.1565	r=0.3526 p=0.3156	**r=0.6970** **p=0.0306**	r=-0.4854 p=0.1875	r=-0.3798 p=0.3094	r=0.04184 p=0.9223	r=0.2298 p=0.5493	r=0.1540 p=0.5968	r=-0.01322 p=0.9659	r=0.4466 p=0.1105	r=0.1545 p=0.5979
**NEU#**	r=0.5951 p=0.0769	r=0.5410 p=0.1099	r=0.6079 p=0.0679	r=0.6000 p=0.0734	r=0.01667 p=0.9816	r=-0.3025 p=0.4267	r=0.1167 p=0.7756	r=0.1187 p=0.7733	r=0.3934 p=0.1652	r=0.2794 p=0.3304	r=0.2264 p=0.4356	r=0.08159 p=0.7812
**NEU%**	**r=0.8835** **p=0.0007**	r=0.5542 p=0.0964	r=0.1757 p=0.6274	r=-0.03647 p=0.9262	r=0.3526 p=0.3520	r=0.4408 p=0.2350	r=-0.07439 p=0.8492	r=-0.3193 p=0.4023	r=-0.2388 p=0.4110	r=0.4370 p=0.1182	r=-0.2527 p=0.3825	r=-0.2945 p=0.3068
**LY#**	r=-0.4796 p=0.1607	r=-0.2173 p=0.5465	r=0.3736 p=0.2876	r=0.6186 p=0.0566	r=-0.3875 p=0.3028	r=-0.6051 p=0.0842	r=0.3610 p=0.3398	r=0.5125 p=0.1583	r=0.4001 p=0.1563	r=-0.2013 p=0.4900	r=0.3363 p=0.2399	r=0.3807 p=0.1793
**LY%**	**r=-0.8763** **p=0.0009**	**r=-0.6350** **p=0.0485**	r=-0.05618 p=0.8775	r=0.1152 p=0.7589	r=-0.3644 p=0.3349	r=-0.4852 p=0.1855	r=0.1946 p=0.6158	r=0.4220 p=0.2579	r=0.2688 p=0.3528	r=-0.4305 p=0.1244	r=0.2703 p=0.3492	r=0.4609 p=0.0972
**MO#**	r=0.1429 p=0.6937	r=0.4730 p=0.1673	r=0.2467 p=0.4921	**r=0.6541** **p=0.0402**	r=-0.06212 p=0.8739	r=-0.3937 p=0.2945	r=0.01312 p=0.9733	r=0.03161 p=0.9357	r=0.1818 p=0.5340	r=-0.1086 p=0.7117	r=0.2819 p=0.3262	r=-0.04924 p=0.8673
**MO%**	**r=-0.6422** **p=0.0453**	r=-0.2692 p=0.4520	r=-0.3414 p=0.3343	r=-0.02432 p=0.9530	r=0.1112 p=0.7758	r=0.08927 p=0.8193	r=-0.3780 p=0.3159	r=0.2579 p=0.6317	r=-0.1375 p=0.6392	r=-0.3494 p=0.2207	r=-0.1344 p=0.6451	r=-0.2642 p=0.3614

WBC, Leucocyte; NEU, Neutrophil; LY, Lymphocyte; MO, Monocyte; #: Peripheral blood cell count; %: Peripheral blood cell percentage.

Correlations at p < 0.05 are shown in red.

### Clustering of CXCR5^+^TIM-3^-^PD-1^+^ T cells by single-cell sequencing analysis

To understand the specific function and mechanism of this cell type, we analyzed single-cell sequencing data from the paranasal sinus mucosa of patients with CRS. As illustrated in **Figure 2**, we unfortunately failed to detect CXCR5^+^TIM-3^-^PD-1^+^ T cells in the single-cell sequencing results.

## Discussion

In the present study, we observed that (1) patients with CRS had significantly increased CXCR5 and PD-1 expression levels in nasal tissue, CD8^+^. and CD4^+^ T cells, respectively, especially in the paranasal sinus mucosa. (2) CXCR5^+^TIM-3^-^PD-1^+^ T cell levels were higher in the paranasal sinus tissues of patients with CRS compared with those of healthy controls. (3) The number and percentage of CXCR5^+^TIM-3^-^PD-1^+^ CD8^+^ T cells in the nasal polyps of patients with CRS were negatively correlated with disease severity. (4) The number of CXCR5^+^TIM-3^-^PD-1^+^ T cells in the paranasal sinus tissues of patients with CRS was negatively correlated with disease duration. (5) The levels of CXCR5^+^TIM-3^-^PD-1^+^ T cells in nasal tissues were positively correlated with blood leukocyte, neutrophil, lymphocyte, and monocyte levels. Our data implies that CXCR5^+^TIM-3^-^PD-1^+^ T cells are involved in the immune process of CRS, thus offering a potential new target for immunotherapy.

Considering the importance of TIM-3, CXCR5, and PD-1 in inflammatory diseases, we first evaluated the expression levels of the three costimulatory molecules on CD4^+^ and CD8^+^ T cells of patients with CRS. We observed that the expression levels of both CXCR5 and PD-1 were significantly increased in the nasal tissues, especially in the paranasal sinus mucosa, of patients with CRS. However, we did not observe elevated TIM-3 expression on T cells in the current study, probably because the number of healthy control mucosa was insufficient to reflect differences between the groups. Our findings indicate that upregulated expression of CXCR5 and PD-1 in localized inflammatory tissues may contribute to the immune dysfunction underlying CRS.

Previous studies have demonstrated that CXCR5^+^CD8^+^ T cell levels appear elevated in viral infections and tumors. CXCR5^+^CD8^+^ T cells heavily express genes associated with hematopoietic stem cell self-renewal and maintenance (25). In addition, this subpopulation has the transcriptional signature of a pool of long-lasting memory cells; its increase implies continued differentiation of terminal cells expressing strong effector functions (7, 10, 26). In mice models, CXCR5^+^CD8^+^ T cells can function as memory stem cells in chronic viral infections and maintain virus-specific CD8^+^ T cell production (11). Our data also demonstrated that the levels of CXCR5+TIM-3-PD-1+ T cells were significantly higher in the paranasal sinus tissues of patients with CRS compared with those in healthy controls. We also observed that CXCR5^+^TIM-3^-^PD-1^+^ T cells were positively correlated with chronic inflammatory and infectious state indicators, i.e., blood leukocyte, neutrophil, lymphocyte, and monocyte levels. Thus, our findings suggest that CXCR5^+^TIM-3^-^PD-1^+^ T cells contribute to the chronic inflammatory and infectious state of patients with CRS.

During immunization, T cells gradually exhibit dysfunction, also known as T cell exhaustion. Exhausted T cells tend to have decreased effector functions, diminished killing capacity, and low proliferative capacity (27, 28). At the same time, the expression of inhibitory receptors – such as PD-1, cytotoxic T-lymphocyte antigen-4, and lymphocyte-activation gene 3 on the surface of the cells is increased (28). The transcriptional signature of CD8^+^ T-cell exhaustion predicts an improved prognosis in a variety of autoimmune diseases (29). Accordingly, inducing exhaustion may be a therapeutic strategy for autoimmune and inflammatory diseases (29). Moreover, we discovered that in CRS, CXCR5^+^TIM-3^-^PD-1^+^ T cell levels decreased with the disease duration. In addition, the CD8^+^ subset of CXCR5^+^TIM-3^-^PD-1^+^ T cells was negatively correlated with disease severity in patients with CRS. This trend was consistent with the association between exhausted T cells and disease prognosis in inflammatory and autoimmune diseases.

In several autoimmune diseases, infections, and tumors, researchers have restored T cell effector function by inhibiting the inhibitory receptor of exhausted T cells, thereby serving as a form of immunotherapy (30, 31). PD-1 targeted immunotherapy is one of the most commonly used immunotherapies for cancer, and PD-1 and T-cell immunoglobulin and ITIM domain may be preferred targets for immune checkpoint inhibitor immunotherapy (32). Interestingly, CXCR5^+^CD8^+^ T cells exhibit a proliferative burst after PD-1 blockade, with a significant increase in further differentiation of this subpopulation (11). CXCR5^+^TIM-3^-^ T cells are robustly multipotent and may serve as precursors for generating effector T cells after PD-1 blockade in advanced disease stages (10, 11). In tumors and viral infections, blocking PD-1 molecules might be able to reverse the exhausted state of such cells and induce their differentiation (7, 11). On the contrary, in chronic inflammatory and autoimmune diseases, the immunotherapeutic approach is likely to be the opposite, aiming to promote the exhaustion of these T cells (29). In conclusion, assuming that CXCR5^+^TIM-3^-^PD-1^+^ T cells are as cytotoxic and stem cell-like in CRS as they are in other diseases, it is possible to intervene by targeting PD-1 to alter the exhaustion state of these cells, thereby modulating the cells’ capacity for sustained killing as well as their ability to self-proliferate and differentiate, and ultimately helping to terminate the continued progression of the disease. This process is expected to be demonstrated in mouse models of CRS. Overall, our findings indicate that CXCR5^+^TIM-3^-^PD-1^+^ T cells could become ideal targets for deregulation in CRS.

Regrettably, this cluster of cells was not identified in the present study when analyzed by single-cell sequencing data.

Notably, the inability of single-cell sequencing to identify this cell subpopulation does not imply that the particular subpopulation was not present in the sequenced samples. Rather, this issue was likely due to the low number and proportion of the cell type, such that mixing the cell type with other numerically abundant cell subpopulations made it challenging to distinguish. As the number and proportion of target cell subpopulations are crucial for single-cell sequencing cluster analysis, if other researchers plan to sequence this cell population, we recommend sorting for immune cells or T cells before sequencing.

Given that the number of cells in this group is small, it poses a great challenge for subsequent mechanism studies. Nevertheless, in the future, further clarifying the role played by CXCR5^+^TIM-3^-^PD-1^+^ T cells in the development of chronic inflammatory and autoimmune diseases by *in vitro* and *in vivo* experiments using genetic modification and other biological means, as well as to investigate the effectiveness of immunotherapies targeting these cells would be of great interest.

The present study had several shortcomings. As the removal of paranasal sinus tissues is not typically necessary for healthy individuals, obtaining a larger sample of healthy control tissues posed challenges. Additionally, some patients in this study had missing data; however, this does not impact the overall trend of the results.

In conclusion, we discovered that the expression of CXCR5 and PD-1 was increased on CD8^+^ and CD4^+^ T cells, respectively, in the nasal tissues of patients with CRS. Moreover, CXCR5^+^TIM-3^-^PD-1^+^ CD8^+^ and CD4^+^ T cell levels were elevated in the nasal tissues of patients with CRS. Furthermore, the CD8^+^ subpopulation was negatively correlated with disease severity in patients with CRS patients. Additionally, CXCR5^+^TIM-3^-^PD-1^+^ T cell levels in nasal tissues were negatively associated with disease duration and positively associated with the chronic inflammatory state of CRS. Our present data suggest that CXCR5^+^TIM-3^-^PD-1^+^ T cells are involved in the chronic inflammatory process underlying CRS and may exert an immune regulatory effect after PD-1 blockade. Consequently, inducing CXCR5^+^TIM-3^-^PD-1^+^ T cell exhaustion may be an effective immunotherapy for CRS. Considering that CRS is a chronic inflammatory disease with autoimmune features, our study has reference value in the understanding of autoimmune diseases and chronic inflammation.

## Data availability statement

The original contributions presented in the study are included in the article/supplementary material. Further inquiries can be directed to the corresponding authors.

## Ethics statement

This study was approved by the Ethics Committee of the First Affiliated Hospital of Soochow University (No. (2023) 056). The studies were conducted in accordance with the local legislation and institutional requirements. The participants provided their written informed consent to participate in this study.

## Author contributions

ZL: Data curation, Formal analysis, Investigation, Methodology, Software, Writing – original draft. ZZ: Formal analysis, Funding acquisition, Writing – original draft. HX: Data curation, Methodology, Resources, Software, Writing – review & editing. NL: Data curation, Formal analysis, Investigation, Writing – review & editing. JL: Funding acquisition, Visualization, Writing – review & editing. QJ: Funding acquisition, Investigation, Project administration, Supervision, Writing – review & editing.
